# Successful Treatment of Leukemic Mature B-Cell Lymphoid Neoplasm with Similar Features to Splenic Marginal Zone Lymphoma Possessing Aberrant Myeloid Markers

**DOI:** 10.1155/2015/181263

**Published:** 2015-10-08

**Authors:** Shinsaku Imashuku, Naoko Kudo, Kagekatsu Kubo, Katsuyasu Saigo

**Affiliations:** ^1^Division of Hematology, Takasagoseibu Hospital, Takasago 676-0812, Japan; ^2^Division of Internal Medicine, Takasagoseibu Hospital, Takasago 676-0812, Japan

## Abstract

In splenic marginal zone lymphoma (SMZL), there are cases that cannot accurately be classified as such because of overlapping morphologic and/or immunophenotypic features. We report here a 76-year-old Japanese female, who showed leukemic B-cell lymphoproliferative disease possessing characteristic features identified for SMZL. The patient was leukemic with white blood cell counts 49,400/*µ*L (abnormal cells, 78.5%) and neoplastic cells were characterized by aberrant expression of myeloid markers with CD19^+^CD13^+^ (64.2%) and CD20^+^CD11c^+^ (25.1%). Considering her history of previous chemotherapy and systemic leukemic phase of the disease, we treated the patient without performing splenectomy, with successful use of a combination of rituximab/bendamustine hydrochloride and of rituximab/cladribine. The patient has been in a complete remission longer than 44 months, with no detectable M-protein.

## 1. Introduction

In 2010, important papers characterizing CD5^−^ and/or CD5^+^ splenic marginal zone lymphoma (SMZL) were published, one in Haematologica and the other in J Clin Exp Hematopathol [1, 2]. Here, we would like to emphasize the significance of mature neoplastic lymphoid cells expressing aberrant myeloid marker as one of the characteristics in SMZL. This lymphoma, consisting of CD5^−^ and/or CD5^+^, is mainly characterized by clonal B-lymphocytes that are positive for CD23 and CD11c in varying percentages. SMZL typically affects elderly patients and is an uncommon indolent B-cell lymphoma causing marked splenic enlargement with CD20-rich lymphoma cells infiltrating blood and bone marrow [3]. However, in cases without palpable splenomegaly, the diagnosis of SMZL is often difficult. In such cases, the expression of myeloid markers such as CD13 and CD11c may help the differential diagnosis of leukemic B-cell lymphoproliferative diseases, though the expression of CD13 is not a salient feature of SMZL.

## 2. Case Report

**Figure 1:**
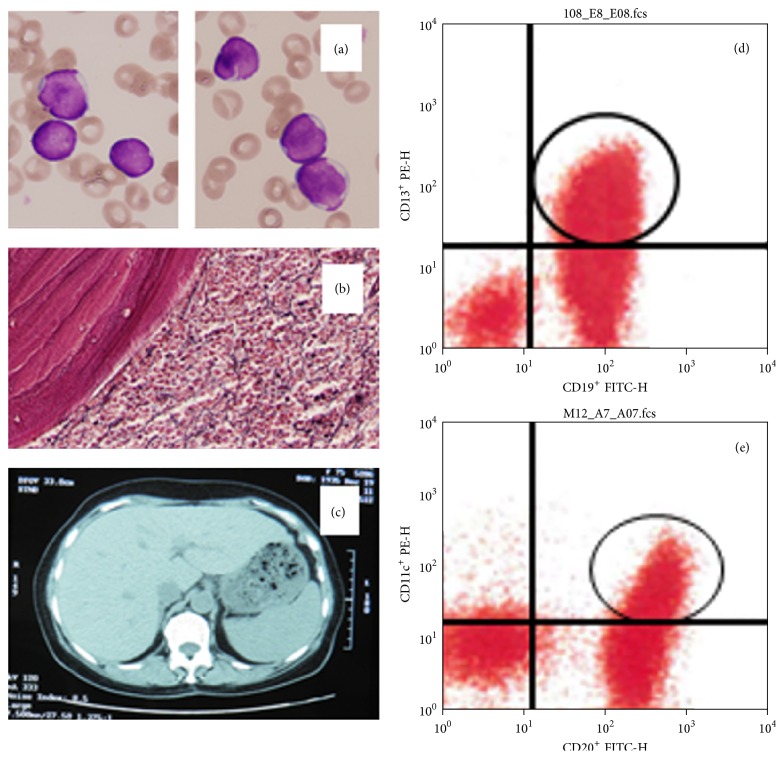
(a) Neoplastic lymphoid cells in the peripheral blood (original magnification ×1,000). (b) Bone marrow core biopsy specimen. BCL-2 positive cells are diffusely infiltrated (×200); CCND1 and EBER in lymphoma cells were negative (data not shown). (c) CT scan of the abdomen showing mild splenomegaly, (d) flow cytometry of peripheral blood showing CD19^+^CD13^+^ neoplastic cells accounted for 64.2% (circled area), and (e) flow cytometry of peripheral blood showing CD20^+^CD11c^+^ neoplastic cells accounted for 25.1% (circled area).

A 76-year-old Japanese female was first noted to be anemic (Hb 7.5 g/dL) in May 2011. Next month follow-up revealed that she had WBC 3,700/*µ*L, Hb 4.6 g/dL, and platelet counts 64,000/*µ*L. Two months later, she was referred to a previous hospital. Over there, although bone marrow aspirations were dry-tap, she was diagnosed to have a leukemic stage of B-cell lymphoma and chemotherapy was started consisting of a pirarubicin/COP regimen; however, the patient refused further treatment after only one course of chemotherapy. When referred to us in December 2011, she was in a partially treated condition and was noted to be leukemic, but she was afebrile and had neither palpable lymph node swellings nor palpable spleen. Leukemic cells in her peripheral blood comprised abnormal small mature lymphocytic cells with a round or slightly irregular-shaped nucleus with a high N/C ratio (Figure 1(a)). Just prior to reinstitution of chemotherapy, her laboratory data were as follows: WBC 49,400/*µ*L (abnormal cells, 78.5%), Hb 7.9 g/dL, platelet counts 33 × 10^3^/*µ*L, LDH 362 (normal values, 122–228) U/L, AST42 (13–37) U/L, ALT (8–45) 40 U/L, total bilirubin 2.2 (0.3–1.3) mg/dL, total protein 7.2 (6.7–8.3) g/dL, albumin 4.4 (4.1–5.2) g/dL, BUN 16.0 (7.8–18.9) mg/dL, creatinine 0.48 (0.45–0.82) mg/dL, uric acid 5.1 (2.5–5.8) mg/dL, CRP 0.60 (0–0.29) mg/dL, HBV negative, HCV negative, and HIV negative. Urinalysis was normal. Other laboratory data showed significantly elevated serum soluble IL-2 receptor (10,300 U/ml; normal 124–466 U/ml), beta-2-microglobulin (3.3 mg/L; 0.9–1.9 mg/L), and high IgM (773 mg/dL; 33–190 mg/dL), but IgG (1118 mg/dL; 870–1700 mg/dL) and IgA (161 mg/dL; 110–410 mg/dL) were within a normal range. Interestingly, IgM-kappa-type M protein was positive, but Bence Jones Protein was undetectable. Bone marrow aspiration was again dry-tap; thus, bone marrow biopsy was underwent, which morphologically showed diffuse infiltration of CD45^+^CD20^+^ mononuclear cells in association with mild myelofibrosis; intrasinusoidal infiltration pattern was not apparent. These lymphoid cells were confirmed to be positive for BCL-2 (Figure 1(b)), but negative for CCND1 and EBER-ISH (data not shown). Mild splenomegaly was shown by CT scan (Figure 1(c)). Further characterization of neoplastic lymphoid cells was only possible using peripheral blood in two occasions with flow cytometry and chromosome analysis. Data showed CD2^+^ (9%), CD3^+^ (18%), CD5^+^ (21%), CD10^+^ (0%), CD19^+^ (94%), CD20^+^ (86%), CD23^+^ (55%), CD25^+^ (20%), CD13^+^ (64%), CD33^+^(0%), CD34^+^ (0%), CD11c^+^ (27%), HLA-DR^+^ (95%), sIgM-kappa^+^ (85%), and sIgM-lambda^+^ (0%). In addition, as double positive subsets, CD5^+^CD20^+^ (5.0%), CD5^+^CD23^+^ (1.0%) (data not shown), CD19^+^CD13^+^ (64.2%), and CD20^+^CD11c^+^ (25.1%) (Figures 1(d) and 1(e)) were noted. In two separate occasions, the same karyotype of 48, XX, +3, der(7)t(1;7)(q21;q22), +18 [1/20] was yielded from bone marrows, which indicates that this was the responsible chromosome abnormality in this case. We treated this case with multiagents' chemotherapy: two courses of rituximab (375 mg/m^2^/dose; day 1)/bendamustine hydrochloride (100 mg/dose, day 2, 3) q 3 weeks, and thereafter with use of repeat 4 courses of rituximab (week 1)/cladribine (5 mg/m^2^/dose, week 2). Eventually, two more courses of rituximab/bendamustine hydrochloride were given all over a total treatment period of 5 months. In terms of treatment toxicity and tolerability, she developed infusion reaction at the first administration of rituximab; however, otherwise, she tolerated the treatment well with grade 1 toxicity for neutrophil counts and for hepatic function determined by the Common Terminology Criteria for Adverse Events v3.0. After the completion of treatment, in April 2012, successful aspiration yielded cellular bone marrow with abnormal cells less than 5% and normal karyotype [46, XX (20/20)]. Blood counts and laboratory data including sIL-2R were all normalized. PET-CT, performed in June 2012, revealed no hot spots in the lymph nodes or in the spleen although spleen was still mildly enlarged. As of August 2015, the patient has maintained a complete remission longer than 44 months, with no detectable M-protein.

## 3. Discussion

This case was characteristic for indolent course in association with lack of B symptoms, absence of lymph node swelling, and mild splenomegaly, but significant leukemic manifestation consisting of abnormal mature small lymphocytic cells with similar features of SMZL and aberrant expression of myeloid markers such as CD13 and CD11c. CD13 is expressed exclusively in myeloid cells while CD11c is a marker for monocytes, macrophages, granulocytes, NK cells, and dendritic cells as well as activated T and B-cells. The definite diagnosis of SMZL could be made by histopathology of splencetomized tissue [1]; however, as described by Kojima et al. [2], three out of their 11 cases did not undergo splenectomy but were diagnosed from characteristic features of leukemic lymphoid cells. In addition, they reported that another 3 patients who underwent splenectomy after combined chemotherapy did not show the marginal zone proliferation pattern any more, which might have disappeared due to chemotherapy effect. Regarding CD5 expression of SMZL, Baseggio et al. summarized 24 cases of CD5^+^ and 42 cases of CD5^−^ SMZL in which they concluded that the immunological profile of the CD5^+^ SMZL in blood was similar to that commonly described for CD5^−^ SMZL [1]. Eleven cases described by Kojima were all CD5^+^ (40–94% positivity) while our case showed negative CD5. Thus, we treated our case under the presumptive diagnosis of CD5^−^ SMZL without performing splenectomy, because she had a history of previous chemotherapy and presented as systemic leukemic phase. In terms of phenotypic characteristics of SMZL, Kojima et al. [2] reported that, out of 11 CD5^+^ cases, 4 showed CD11c^+^ and 5 showed CD13^+^. On the other hand, Baseggio et al. [1] reported that CD11c was positive in only 2/24 CD5^+^ cases (8%) and they did not mention the CD13 positivity. Thus, we first characterized here neoplastic lymphoid cells with CD5^−^CD19^+^CD13^+^ as well as CD5^−^CD20^+^CD11c^+^ in our case. In SMZL, the karyotypic changes such as 7q deletion, trisomy 3, and trisomy 18 were noted [1]. Exactly the similar karyotype was yielded in two separate occasions in our case, although abnormal karyotype was [1/20] each time. Thus, we suspected that our case highly likely had SMZL and the possibility of mantle cell lymphoma was ruled out from negative CCND1 staining and that of hairy cell leukemia from cell morphology and barely detectable CD25; however, we missed a chance by applying FISH procedures in the process of diagnosis to differentiate SMZL from other leukemia/lymphomas. Other diagnostic possibilities may still exist, but we weighed more on the presence of CD19^+^CD13^+^ and CD20^+^CD11c^+^ neoplastic cells in making the diagnosis of this case. It seems that most plausible hypothesis is that this was a case of progression to leukemia of monoclonal B-cell lymphocytosis expressing aberrant myeloid markers, consistent with features displaying many similarities with the SMZL [4–6]. Regarding the prognostic factors of SMZL, presence of M-protein component, an elevated beta-2-microglobulin level, and WBC > 20,000/*µ*L (lymphocytes > 9,000/*µ*L) may predict a poor prognosis [3]. Our patient fulfilled all of these criteria. In terms of treatment of SMZL, effectiveness of rituximab was well recognized [7]. In addition, purine analogues alone or in association with rituximab are recommended to be a valid therapeutic choice for SMZL [8, 9]. The reason we chose our treatment regimens consisting of alternate rituximab/bendamustine and rituximab/cladribine was that the patient had high-risk features [3] and was suspected to have refractory disease due to (1) partially treated, (2) clearly in a leukemic phase, and (3) aberrant CD13 expressing mature B-cell lymphoma. We also thought that the patient needs short-term aggressive therapy to obtain a cure, although initially we had difficulty to predict the eventual outcome. Fortunately, she responded well to the rituximab/bendamustine and cladribine regimens and has attained a long-term complete remission. In conclusion, characteristic phenotypic/karyotypic features may help in assuming the diagnosis of SMZL or SMZL-like mature B-cell lymphoid neoplasm from the blood or bone marrow examinations alone without splenectomy and promise the patient a good outcome with use of appropriate chemotherapy.

## References

[1] Baseggio L., Traverse-Glehen A., Petinataud F. (2010). CD5 expression identifies a subset of splenic marginal zone lymphomas with higher lymphocytosis: a clinico-pathological, cytogenetic and molecular study of 24 cases. *Haematologica*.

[2] Kojima M., Sato E., Oshimi K. (2010). Characteristics of CD5-positive splenic marginal zone lymphoma with leukemic manifestation; clinical, flow cytometry, and histopathological findings of 11 cases. *Journal of Clinical and Experimental Hematopathology*.

[3] Thieblemont C., Felman P., Berger F. (2002). Treatment of splenic marginal zone B-cell lymphoma: an analysis of 81 patients. *Clinical Lymphoma*.

[4] Mowery Y. M., Lanasa M. C. (2012). Clinical aspects of monoclonal B-cell lymphocytosis. *Cancer Control*.

[5] Kalpadakis C., Pangalis G. A., Sachanas S. (2014). New insights into monoclonal B-cell lymphocytosis. *BioMed Research International*.

[6] Kostopoulos I. V., Paterakis G., Papadimitriou K., Pavlidis D., Tsitsilonis O. E., Papadhimitriou S. I. (2015). Immunophenotypic analysis reveals heterogeneity and common biologic aspects in monoclonal B-cell lymphocytosis. *Genes Chromosomes and Cancer*.

[7] Kalpadakis C., Pangalis G. A., Dimopoulou M. N. (2007). Rituximab monotherapy is highly effective in splenic marginal zone lymphoma. *Hematological Oncology*.

[8] Cervetti G., Galimberti S., Sordi E. (2010). Significant efficacy of 2-CdA with or without rituximab in the treatment of splenic marginal zone lymphoma (SMZL). *Annals of Oncology*.

[9] Bennett M., Schechter G. P. (2010). Treatment of splenic marginal zone lymphoma: splenectomy versus rituximab. *Seminars in Hematology*.

